# Prevention and early intervention of depression in young people: an integrated narrative review of affective awareness and Ecological Momentary Assessment

**DOI:** 10.1186/s40359-021-00614-6

**Published:** 2021-08-16

**Authors:** Joanne R. Beames, Katarina Kikas, Aliza Werner-Seidler

**Affiliations:** Black Dog Institute, University of New South Wales, Sydney, NSW Australia

**Keywords:** Affective awareness, Ecological momentary assessment, Youth, Depression, Prevention, Early intervention

## Abstract

Depression has a devastating impact on young people around the world. This impact is pervasive and long-long lasting, still causing havoc as young people transition into adulthood. Prevention and timely early intervention efforts are necessary to lessen the disease burden of depression in young people. There is some evidence for the effectiveness of existing prevention and early intervention programs when delivered early. However, there is no consensus on what the active ingredients of these programs are. Identifying these ingredients is important because focusing on those that have maximal benefit will help to minimise the costs and resourcing of (already intensive) therapeutic approaches. We explored whether, and in what circumstances, affective awareness (i.e., knowing how one feels) is a candidate for protecting against and decreasing vulnerability to depression in young people. We specifically looked at how Ecological Momentary Assessment methods (EMA) were used to measure and/or increase affective awareness. We integrated three streams of data, including narrative review findings, perspectives from young people and psychologists, and publicly available information online (e.g., discussion forums). Across data sources, we found converging evidence that affective awareness is important in the prevention and early intervention of depression although there were considerable gaps in knowledge. More work needs to be done with young people and their health care teams to build affective awareness in the right way, at the right time, with individual differences in mind. The identification of how EMA can be best incorporated into young people’s lives to facilitate these outcomes is also needed.

**Supplementary Information:**

The online version contains supplementary material available at 10.1186/s40359-021-00614-6.

## Introduction

Depression is a leading cause of disability worldwide, with debilitating effects on psychosocial, physical, and emotional functioning [1, 2]. The onset of depression is typically around mid-to-late adolescence, with earlier onset associated with a more severe clinical course [3, 4]. Given the impact caused by depression, together with limited success of treatment approaches [5], the prevention and early intervention of depression is imperative. There is evidence supporting psychological prevention approaches during adolescence [6–8].

Knowledge about psychological processes primarily responsible for therapeutic prevention or change (i.e., active ingredients) is lacking [9]. Focusing on the active ingredients that work will lead to the development of more effective and efficient interventions that enhance mental health outcomes for the individuals that use them [10]. In 2020, The Wellcome Trust Mental Health Priority Area launched its first active ingredients commission to explore aspects of interventions that are most effective for preventing and treating anxiety and depression in young people. The commissioned review presented here builds the case for affective awareness—a foundational skill that can protect young people (aged between 14 and 20 years) against depression and improve their emotional health. We used creative methods to emphasise lived experiences and move beyond the scope of traditional scientific reviews, which are not without limitation [11]. There are many benefits of including lived experience perspectives. For example, such perspectives can produce better quality research by improving methodological sensitivity, validity of results, and relevance to the target population [12, 13]. Our integration of data enables comparison of what is known from research, what is done in practice, and what young people want in their mental health care.

### Active ingredient: affective awareness

Affective awareness is the ability to perceive, describe, understand, and differentiate emotions and moods [14, 15]. Imagine that Talia is feeling sad. With affective awareness, she will be able to identify her experience as sadness, rule out other emotions (e.g., regret, calm), describe her internal sensations (physical and cognitive), and understand her behavioural responses to the emotion (e.g., social withdrawal). Affective awareness is developed early in life [15] and is a building block for other emotional processes such as emotion regulation [16]. This means that if Talia is aware of her feelings, she will be able to communicate her emotional state (e.g., I feel sad), reach out for help, and implement helpful coping/regulation strategies (e.g., perspective taking, focusing on an upcoming holiday). Strategy selection is important because those that are effective for overcoming sadness might differ from those that are effective for other negative emotions like guilt. The key message is that if young people do not know they feel sad or down, it will be difficult for them to respond to those feelings in a helpful way.

### Affective awareness in youth depression

Affective awareness (or lack thereof) is putatively involved in the development and maintenance of depressive symptoms between 7 and 24 years of age [17, 18]. There are multiple and complex reasons why adolescents have an increased vulnerability to depression. Young people undergo natural biological maturation processes that often produce changes in moods and emotions, and their regulation skills are not fully developed [19]. For example, young people who are prone to depression are less aware of their emotions, have difficulty understanding them, and have limited capacity to tolerate them (e.g., [20–22]). Young people also have a greater tendency to respond to stress with negative affectivity [23]. Adolescence is therefore a critical time whereby affective skills can be developed and shaped to improve emotional health.

### Measuring (and increasing) affective awareness using EMA

In clinical practice, affective awareness is typically measured with pen-and-paper or digital mood monitoring tools where the individual is asked to reflect on their emotions and activities through the week [24]. In research studies, affective awareness is typically measured as a self-report trait characteristic [22] or, more recently, through ecological momentary assessment (EMA; [25]). EMA is an effective way to study affect and depression-related phenomena [26]. It allows individuals to repeatedly report their emotions and moods in real-time. Completed using technology, including smartphones, users typically receive one or more prompts each day to report how they are feeling and the context around that feeling (e.g., what they are doing/thinking). The advantages of EMA over traditional methods include a higher level of temporal detail that captures real-time changes in experiences, ecological validity, and reliability.

EMA provides an indirect measure of affective awareness; an individual must be aware of (as well as label and communicate) an emotional experience to be able to report that experience. One study found a strong positive correlation between a self-report emotional awareness scale and EMA, suggesting that the two types of assessment measure a similar construct [27]. There is also *some* (albeit mixed) evidence that EMA itself can change the emotional experiences and mental health symptoms that it is measuring (i.e., assessment reactivity). One study found that EMA of positive and negative affect over 6-weeks may have a beneficial short-term effect on depressive symptoms in clinically depressed adults [28]. A systematic review concluded that mobile mood-monitoring applications may reduce depressive symptoms in young people aged 10–24 years by increasing affective awareness [29]. Together, these findings indicate that EMA can be used to measure emotional experiences and can also be used as a brief intervention with clinical outcomes. The general EMA methodology underpinning both approaches is comparable.

There are other psychological treatment approaches that aim to influence affective awareness. One evidence-based approach is Mindfulness-Based Cognitive Therapy (MBCT). MBCT aims to cultivate awareness of thoughts, feelings, sensations, and feelings in the present moment, and develop new ways of relating to thoughts and feelings. It is a multi-component approach that is resource intensive (time and financial cost). Emerging evidence suggests that MBCT can have beneficial effects for young people with and without depression, but robust causal studies with insights into mechanisms are lacking [30]. This means that the extent to which changes in affective awareness drive treatment effects is unclear. The current review will focus exclusively on EMA rather than treatment approaches such as MBCT. This approach will allow us to determine the relationships between EMA, awareness, and depression symptoms in young people.

### Research gap

Despite the potential for EMA in measuring and changing affective awareness or depression-related phenomena in youth, research is lacking [31]. EMA studies with youth have rarely examined associations between momentary fluctuations in affective experience, awareness, and clinical outcomes [32]. Further, it is unclear what young people and mental health professionals think about EMA and how it can be used in clinical practice and daily life.

### Aims

The aims of this review are to assess: (1) whether EMA increases affective awareness and reduces depression symptoms; (2) whether EMA can identify depression risk; and (3) stakeholder perspectives about affective awareness and using EMA in daily life, in and outside of the therapy context. Aim 3 focuses on what young people think about affective awareness in relation to depression and mental health, whether they use or would be likely to use EMA to facilitate awareness, as well as their reasons why. An embedded aim is to identify the gaps between research, clinical practice, and what young people are doing in their daily lives.

## Overview of methodological approaches

To address these aims, we used a creative approach that combined a narrative review with qualitative methods that drew from stakeholder perspectives. The stakeholders included young people, some with lived experiences of depression, and psychologists. We also searched websites and publicly available online discussion boards/forums for affective awareness, depression, and mood monitoring. The novelty of our approach lies within the integration and equal weighting of data sources, which was consistent with the guidelines outlined by the 2020 Wellcome Trust active ingredients commission. The value of integrating empirical review and qualitative data was in being able to discover a full range of perspectives, highlighting gaps between research, practice, and lived experience. Integration also provided insight about what young people are looking for and how researchers and clinicians can work together to address their needs. Our approach offers new insights that would not otherwise be determined from a traditional scientific review, representing a key strength and shift in how evidence and experience can be incorporated in the future.

Our approach builds on current knowledge by: (1) considering phone-based EMA as an intervention and/or measurement tool; (2) focusing on prevention *and* early intervention; (3) exploring different study designs; (4) incorporating lived experience perspectives; and (5) integrating cross-disciplinary insights from clinical psychology and emotion research. We also drew inferences from treatment studies where young people have been diagnosed with depression.

### Narrative review

Electronic databases including PsycINFO, PubMed, EMBASE, Google Scholar and CINAHL were searched between July 2020 and August 2020 using key search terms, including (but not limited to): “awareness” or “self-awareness”, and “monitor” or “track” or “experience sampling” or “ecological momentary assessment” or “EMA” or “label” or “diary”, and “youth” or “adolescents”, and “depress” or “mood” or “emotion”. We also hand-searched the reference lists of relevant articles, and systematic reviews [29, 31] and meta-analyses [22] were reviewed to identify any peer-reviewed evidence that might have been missed in the literature search. The initial inclusion criteria were young people aged between 14 and 20, sub-clinical or non-clinical samples, a measure or report of affective awareness (i.e., self-report or verbal report), phone-based EMA methodology, and a measure of depressive symptoms. We focused on phone-based EMA because data collection can be relatively unobtrusive and more young people can be reached at any one time regardless of their location. The low-cost, high availability, and in-built flexibility of phone-based EMA could overcome barriers to traditional healthcare, contributing to more sustainable monitoring tools that can be delivered at scale. As such, quantitative studies that focused on young people and phone-based EMA were included (rather than pagers, emails, websites etc). There was limited availability of research evidence after our initial search. We therefore expanded our search to include studies that used clinical populations, young people and adults, and other indices of emotional awareness (e.g., emotional clarity, differentiation, and emotional intelligence). Additionally, if mood monitoring was combined with another psychological therapy (e.g., mood monitoring plus cognitive behavioural therapy), we included it in our review. There were no restrictions on publication year. Our search revealed 11 relevant articles (see Table 1). In the following sections, unless otherwise noted, the terms adolescent, young people or youth refer to individuals aged 14–20.

**Table 1:** Study details for the literature review

Study & country	EMA component/ intervention	Design	Age	*N*	Mental health status	Timing	Outcomes	Summary of results
*Youth (7–24)*								
Reid et al. (2011) Australia	App-based EMA monitoring of various states, including mood. Used as an intervention	RCT	14–24	114	Sub-clinical, mild-moderate mental health concerns	IP	DASS ESA	Self-monitoring, via EMA, increased emotional self-awareness, but had no effects on depression
Kauer et al. (2012) Australia	App-based EMA monitoring of various states, including mood. Used as an intervention	Secondary analysis of Reid et al. (2011)	14–24	114	Sub-clinical, mild-moderate mental health concerns	IP	DASS ESA	Self-monitoring, via EMA, had an indirect effect on depressive symptoms via emotional self-awareness. The direct effect was not significant
De Vuyst et al. (2019) Belgium	App-based EMA mood monitoring. Used as an intervention	Pre-post	18–24	90	Non-clinical	UP	PHQ-9	EMA mood monitoring had no effect on affective experience or depressive symptoms
Silk et al. (2011) USA	EMA mood monitoring (phone calls). Used as a measurement tool in a treatment trial, to explore daily emotion dynamics	Quasi	7–17	79	Clinical (DSM-IV diagnosis of MDD required for inclusion into experimental group)	N/A	PANAS-C	Depressed participants reported more intense negative emotions, including greater sadness, anger, and nervousness, as well as a lower ratio of positive to negative affect
Forbes et al. (2012) USA	EMA mood monitoring (phone calls). Used as a measurement tool in a treatment trial, to predict response & course	Cross-sectional	8–16	66	Clinical (K-SADS-PL diagnosis of MDD required for inclusion)	N/A	PANAS-C	Higher negative affect and a lower ratio of positive to negative affect were related to a slower rate of decline of clinical severity during treatment
Sheets & Armey (2020) USA	App-based EMA mood monitoring. Used as a measurement tool, to explore daily emotion dynamics	Quasi	18–22	108	Clinical (DSM-IV diagnosis of MDD required for inclusion into experimental group)	N/A	Negative affect	Currently depressed youth reported greater average negative affect, and greater increases in negative affect to recent perceived stress, than the other two groups
Van Roekel et al. (2016) *Study 1* Netherlands	App-based EMA mood monitoring. Used as a measurement tool, to explore daily emotion dynamics	Cross-sectional	13–16	284	Non-clinical	N/A	BSI-AV Positive affect	Depressive symptoms were related to lower mean positive affect and higher variability in positive affect regardless of sex
Yen et al. (2020) USA	EMA mood monitoring (text messages) delivered as part of FTF STEP	Pilot RCT	12–18	52	Clinical (psychiatric inpatients, admitted for suicide attempt or ideation)	T	mDES BDI-II	Parent ratings of depression were lower for youth in the intervention condition compared to the control at 3 months but not at 6 months post-treatment. No group differences for positive and negative emotions
Bai et al. (2020) USA	App-based EMA mood monitoring delivered as part of FTF mindfulness. Used as a measurement tool in a treatment trial	RCT	18–19	52	Non-clinical	UP	DISE Negative emotion	All participants reported more negative emotions when they experienced greater than usual levels of family stress, but the association was weaker for those in the mindfulness condition
*Youth and Adults (13–69)*								
Bakker & Rickard (2018) Australia	App-based EMA mood monitoring. Used as an intervention	Pre-post	13–69	234	Broad community sample. Analyses by sub-clinical (≥ 15 on PHQ-9 or GAD-7; or > 10 on both) and non-clinical	UP	ESAS-R PHQ-9 App engagement scale	EMA engagement predicted decreases in depression. These effects were mediated by increases in emotional self-awareness, but only for the sub-clinical subset
Fitzpatrick et al. (2017) USA	App-based CBT with EMA mood monitoring	RCT	18–28	70	Sub-clinical (self-identified symptoms; 46% mod-severe or severe depression at baseline)	IP	PHQ-9 PANAS Qualitative reports of affective awareness	Intervention reduced depression symptoms and participants qualitatively reported a greater amount of affective awareness

**EMA Component/Intervention**—EMA = ecological momentary assessment, FTF = face-to-face, CBT = cognitive behavioural therapy, STEP = Skills to Enhance Positivity Program; **Study Type**—RCT = Randomised controlled trial; **Mental Health Status**—DSM-IV = Diagnostic Statistical Manual, Fourth Edition, K-SADS-PL = Kiddie Schedule for Affective Disorders and Schizophrenia—Present and Lifetime Version, MDD = Major Depressive Disorder; **Timing**—Timing of the intervention, IP = Indicated Prevention, which involves individuals who have sub-clinical symptoms but do not meet criteria for a clinical disorder (can also be considered early intervention; UP = Universal Prevention, which involves all individuals in a population irrespective of risk or symptom levels; T = Treatment; **Outcomes** that are relevant for the current review—DASS = Depression Anxiety Stress Scales, ESA = Emotional Self-Awareness Scale, PHQ-9 = Patient Health Questionnaire-9, PANAS-C = Positive and Negative Affect Schedule for Children, BSI-AV = Brief Symptom Inventory-Adolescent Version, mDES = modified Differential Emotions Scale, BDI-II = Beck Depression Inventory, DISE = Daily Inventory of Stressful Events, ESAS-R = Emotional Self-awareness Scale Revised, PANAS = Positive and Negative Affect Schedule, GAD-7 = Generalised Anxiety Disorder 7

Nine studies targeted a youth sample. An additional two included adults and youth. Three studies examined whether affective awareness is related to depression in youth by using EMA as an intervention, and by linking indirect momentary reports to self-report state measures [33–35]. One study used EMA as an intervention and measured depression and other emotional experiences but did not confirm levels of awareness using a self-report measure [36]. Two examined EMA as a component of a broader psychological intervention (e.g., CBT) but did not isolate effects [37, 38]. Another five used EMA as a measurement tool, one in the context of a universal prevention trial [39], another in the context of a treatment trial [40], and three in the context of daily life [41–43]. The literature is sparse with varying study designs, ages, and EMA methods (e.g., signalling type and duration).

### Stakeholder perspectives

Stakeholders were: (1) young people between 16 and 20 years^1^who had experience with or knowledge about mood monitoring; and (2) psychologists who had experience providing or recommending mental health care to young people. A combination of convenience and snowball sampling methods were used to recruit young people and psychologists into the study between July and August 2020. Strategies included online media advertisements (e.g., Black Dog Institute’s website, Facebook, Twitter, and Instagram), and contacting existing professional networks and peak clinical bodies (e.g., Australian Clinical Practice Association [ACPA]).

### Online surveys

Participants provided personal information through a self-report online survey. Young people reported their age, gender, mental health history (*“*Have you ever experienced a mental health problem or been diagnosed with a mental illness?” [Yes, No, I’m not sure]), psychological treatment (“Have you ever received psychological therapy for depression or low mood? [Yes, No, I don’t know], experience with mood monitoring (including digital tools; e.g., “Have you ever used mood monitoring techniques to track how you are feeling in day-to-day life?” [Yes, No, I don’t know]), and mental health (Depression Anxiety Stress Scales [DASS-21], Kessler Scale [K6], Emotional Self-Awareness Scale-Revised [ESA-R]). All mental health measures have been used and validated with young people and have strong psychometric properties [33, 44, 45]. Psychologists provided demographic information, qualifications, employment, and use of mood monitoring in clinical practice. This information was used to contextualise the stakeholder perspectives, expanded upon below.

#### Interviews and focus groups

We conducted six focus groups with young people and individual interviews with psychologists. Questions were asked in a semi-structured format and discussions were prompted using specific, open-ended questions (Additional file 1: Appendix A). Topics included: awareness of emotions and moods, understanding and use of mood monitoring, understanding and use of EMA, mood monitoring in psychological therapy, views about depression, and gaps in psychological treatment.

#### Procedure

Interested stakeholders participated between July 2020 and September 2020. Online surveys for young people and psychologists were administered through Qualtrics (2017). Stakeholders provided electronic informed consent, followed by the questionnaires, which took between 10 and 15 min to complete. In line with the Australian National Statement on Conduct in Human Research [46], young people 16 and above provided informed consent; parental/guardian consent was not required. Focus groups (60–90 min) or interviews (30–60 min) were then held using video conferencing software (Zoom 2011) at a time preferred by the stakeholders. They were facilitated by the chief investigator (JRB) and/or the project officer (KK), audio-recorded, and later transcribed. Young people and psychologists were reimbursed for their time at a rate of $20AUD/h via electronic gift card. Survey and focus group/interview procedures received ethical approval from the University of New South Wales Human Research Ethics Committee (HC200475).

### Brief review of publicly available information and online forums

We reviewed information from known websites, forums, and membership lists (QuantifyMe, Reddit, Beyond Blue, ACPA listserve, Black Dog Institute e-Mental Health in Practice [eMPRac] online forums) for conversations about how young people and psychologists use mood monitoring tools. Key search terms included “mood monitor” and “mood tracking”. We then performed google searches using the terms “depression forum” and “young people forum” to identify youth mental health forums that allowed posts about user experiences with mood monitoring. We explored and extracted queries to get a sense of what tools were being recommended in the public domain (Additional file 1: Table S1 in Appendix B).

### Linking the review with qualitive inquiry

The review informed the topics explored in our qualitative inquiry, with findings from the inquiry acting to evaluate and shape our search terms. Focus groups were also used to ask young people about the legitimacy of our online forum searches and their opinions about the online recommendations. Using a dynamic and integrated approach, information gathered from each source was then used to shape the approach and interpretation of the other sources. This approach increased the rigor and breadth of our methods while also positioning the stakeholder perspectives at the forefront of the review.

## Data analysis

Descriptive statistics were used to characterise the stakeholder samples. Reflexive thematic analysis, according to Braun and Clarke’s six-stage guidelines, were used for qualitative data [47]. Using a deductive approach, analysis involved an iterative process of reading and coding responses to extract overarching themes that mapped onto our research questions. Initial coding was conducted by KK. Refinement of codes and generation of higher-order themes was conducted by KK and JRB. Research questions included: (1) What is the role of affective awareness in depression (and mental health more broadly)?; (2) What are stakeholders’ experiences of mood monitoring, including preferences, benefits, and harms?; and (3) Is EMA a useful tool to understand and improve affective experiences?

## Characteristics of the stakeholder samples

See Table 2 (and Additional file 1: Table S2 in Appendix C). Twenty-four young people, with a mean age of 17.67 (*SD* = 1.34), responded to the survey and 18 contributed to focus groups. Most were born in Australia (75%), identified as female (66.7%), and attended secondary school between years 10–12 (75%). None identified as Aboriginal or Torres Strait Islander. Just over half had been diagnosed with a mental health illness (58.3%), of which 85.7% had received psychological therapy for depression. Just over half had used mood monitoring techniques (54.2%), with most recommendations made by a psychologist during therapy (61.5%). Most young people were open to using mood monitoring in the future to increase affective awareness (87.5%).

**Table 2:** Mental health characteristics of the young people

Measure	Young people	
	*M* (*SD*)	Clinical ranges
*K6*	18.00 (5.3)	Moderate range
*DASS-21*		
Stress	18.42 (8.96)	Average range
Depression	18.67 11.81	Average range
Anxiety	14.25 (9.41)	Moderate to severe range
*ESA-R*	61.83 (16.40)	N/A

Average scores on the K6 indicated that psychological distress was within the moderate range (*M* = 18.00, *SD* = 5.3), with 41.7% indicating the presence of probable mental illness. On the DASS, average scores for stress (*M* = 18.42, *SD* = 8.96) and depression (*M* = 18.67, *SD* = 11.81) were within the moderate range, and anxiety was within the moderate to severe range (*M* = 14.25, *SD* = 9.41). For depression, 66.6% were in the clinical range (i.e., moderate to extremely severe). The average score on the ESA-R scale was 61.83 (*SD* = 16.40), with higher scores indicating higher levels of awareness.

Five female clinical or registered clinical psychologists, with a mean age of 35.00 (*SD* = 8.72) and 6.60 years (*SD* = 7.55) of experience working with young people, also contributed their perspectives. None identified as Aboriginal or Torres Strait Islander, and three were born in Australia. Two indicated that they had received specific training in adolescent mental health. All reported recommending mood monitoring as part of treatment for youth depression at least some of the time. Four indicated that monitoring results impacted their therapeutic approach (one being unsure). Only one had used EMA as a mood monitoring tool in treatment for youth depression.

## Results

### Setting the scene with stakeholder insights

#### How aware are young people of how they feel?

Most interviewed young people were not always aware of their feelings, and older age was related to increased awareness. Negative emotions (e.g., anger, sadness) were reported to be particularly noticeable and memorable, especially when they were high intensity. Some reported that basic (vs complex) emotions were easier to identify. Two reported that being aware of what *others* were feeling could guide social interactions. Most wanted to increase their awareness of both negative and positive emotions.

Psychologists had mixed perspectives about how aware young people were of their emotions. One also clarified that socio-economic circumstances and family environments likely contribute to awareness level, because of norms around emotions, modelling, and links with general emotional skills.

#### What is the role of affective awareness in depression?

All young people and psychologists thought that a lack of awareness contributes to depression. Most young people described this in terms of contributing to a problem that already existed, rather than increasing vulnerability. One psychologist also noted that only being aware of one emotion, rather than the full range, could be a risk factor for depression. Another suggested that learning affective awareness skills from an earlier age, from parents or in primary schools, facilitated prevention.

### Integrated perspectives from research and stakeholders

#### Does EMA increase affective awareness and reduce depressive symptoms?

##### EMA as a stand-alone intervention

Using an indicated prevention approach, one randomised controlled trial (RCT) used EMA as an intervention with a subclinical youth sample between 14 and 24 years of age [34]. Youth in the experimental group monitored a range of experiences including mood; those in the control monitored daily activities. Monitoring for 2–4 weeks increased affective awareness in the experimental group, but depression decreased similarly for both groups at the immediate post-test and 6-week follow-up. In a secondary mediation analysis of this data, Kauer and colleagues found that EMA monitoring decreased depressive symptoms *through* increased awareness for both groups [35]. The strength of this indirect association was larger for the experimental than the control group, and EMA did not directly decrease depressive symptoms. A universal uncontrolled pre-post study with ages across the lifespan (13–69 years) evaluated the effectiveness of MoodPrism (a dedicated mood monitoring app) over 30-days [48] and assessed whether awareness mediated the effects on depression [33]. Perceptions that the app was more (vs less) engaging predicted decreases in depression (for both sub-clinical and non-clinical samples). The effects were mediated by increases in emotional self-awareness, but only for those who were sub-clinically depressed at baseline. Another universal uncontrolled pre-post study with university students (18–24 years) found a different pattern of results [36], with no differences between EMA-group (i.e., positive, negative, or neutral affect) on affect or depressive symptoms. However, there was a time effect for depressive symptoms, with symptoms reducing from pre to post, but increasing from post to the 1-month follow-up. Together, these studies indicate that EMA decreases depressive symptoms by increasing awareness, with effects potentially greater for indicated samples.

Stakeholder insights generally aligned with the review results. Most stakeholders thought that EMA would increase awareness of feelings, and that this awareness could be useful for prevention and/or early intervention. The rationale was that awareness helped to keep track of emotions, identify triggers, and understand why emotions occurred. Some young people were doubtful about whether they would use EMA to monitor their feelings when they were feeling well. This suggests a preference for indicated prevention/early intervention rather than a universal or selective prevention approach (i.e., in the absence of symptoms).

##### EMA as part of a broader intervention

Two RCTs examined EMA as part of a broader psychological intervention. Using an indicated prevention approach, one compared a two-week iCBT intervention (Woebot) with embedded mood monitoring to a control group in a university sample (18–28 years) with self-reported depressive symptoms [37]. Participants in the Woebot group reported reduced depression two weeks post-intervention, and qualitatively reported increased affective awareness. In a pilot RCT with adolescents (12–18 years) admitted to a psychiatric inpatient unit for suicidality, a skills-based positive affect and positive psychology intervention (STEP) plus mood monitoring (EMA) was compared to treatment-as-usual [38]. STEP + EMA was associated with a greater reduction in parent ratings of youth depression compared to treatment as usual 3-months post-intervention. There were no group differences across time for self-reported depression or positive and negative emotions, although engagement in the monitoring was high. These two studies indicate there could be some benefit in EMA approaches as part of broader interventions in the treatment of depression.

The stakeholder psychologists indicated that that they conceptualised EMA as part of a broader intervention rather than a standalone intervention. EMA was considered to facilitate the identification of mood, and how mood changes over time, as well as raise the alarm that their client might be struggling and facilitate help-seeking. A few psychologists and young people also noted that although awareness was necessary, and could lead to improvements by itself, other explicit strategies to improve low mood were needed. Similarly, one psychologist suggested that EMA monitoring could increase emotional intelligence, with knock-on effects for prevention and regulation of mood.

#### Can EMA identify depression risk?

Other EMA studies have not directly measured affective awareness but provide information about links between daily emotion and depressive symptoms. In one RCT, healthy university students (18–19 years) were assigned to an 8-week mindfulness intervention or a waitlist control [39]. A subset was randomly selected to complete EMA measures in short bursts (10-days each) before, during, and after the intervention. Average levels of negative emotion did not differ between condition or burst. All groups reported more negative emotion when faced with family stress, but the effect was weaker among intervention participants. Another cross-sectional study with healthy school students (13–16 years) used a smartphone program (MyExperience) to measure positive affect over 6 days [43]. Depressive symptoms were related to lower mean positive affect and higher variability in positive affect. Using the same program over 2-weeks, a quasi-experiment found that currently depressed with university students (18–22 years) reported greater average negative affect, and greater increases in negative affect to recent perceived stress, than remitted and healthy students [42].

Two treatment studies with depressed youth (7–17 years) measured positive and negative affect using phone-based EMA. The first used a quasi-experimental approach and delivered EMA over 8-weeks [41]. Compared to healthy controls, young people with depression reported more intense negative emotions (e.g., anger, sadness), as well as a lower ratio of positive to negative affect. The second study used a cross-sectional design and EMA to gather baseline data over four days before depressed youth received one of three treatment protocols [40]. Higher negative affect and a lower ratio of positive to negative affect at baseline were related to a slower rate of decline of clinical severity during treatment. Overall, these studies show that increased variability in negative emotion could be a key risk factor for youth depression, and real-time assessment of mood can provide important information about outcome and course of treatment.

Stakeholder insights converged with the research findings. For both stakeholder groups, in-the-moment functionality of EMA was thought to be a major benefit in prevention and early intervention strategies. It was reported that increasing awareness of emotions and understanding patterns in real-time helps to identify triggers early and facilitate action, thus reducing risk of depression onset and severity.

### Gaps between research, clinical practice, and what young people want

The narrative review did not provide insight into preferences for EMA, barriers to use, potential harms of affective awareness, or the types of individuals that might benefit most from increased awareness. Identifying these characteristics is important because they speak to whether young people will use EMA, who it might be more effective for, and appropriateness. The stakeholder insights filled this gap.

#### Stakeholder experience and preferences for mood monitoring, with an emphasis on EMA

About half of the young people interviewed had not used any form of mood monitoring, although most had heard about it and could foresee benefits. For those that did have experience, methods used included journaling, app-based EMA monitoring (e.g., Daylio), and pen-and-paper monitoring. When apps were introduced as part of psychological therapy, the rationale was to overcome barriers with traditional pen-and-paper methods such as forgetting to monitor and difficulties recalling retrospectively. Individual differences, flexibility, and autonomy were key factors for engagement.

There were uncertainties and variation around the ideal functions of EMA. Most young people thought that identifying emotional content, intensity, *and* context was important. One young person thought that colours or imagery would accurately represent mixed emotions. Another preferred to report potent experiences ad hoc, rather than in response to a pre-determined prompt.

All psychologists reported using pen-and-paper mood monitoring in their practice. Most used it in the earlier phases of treatment as a between session home task. Common themes were that approaches were chosen based on usability and appropriateness for each young person, and that data was used to paint a broad picture of emotional experience. At the time of interview, none had used app-based or EMA tools although all thought that EMA would be useful. They thought EMA-apps could overcome barriers to traditional monitoring approaches, with benefits including increased uptake, simplicity, and accuracy.

The interest in EMA and app-based tools expressed by the stakeholders converged with our search of publicly available information. Although there are many websites giving recommendations for mood monitoring apps [49], they should be viewed with caution because few apps are designed specifically for young people and have been evaluated in research trials. Overall, we found evidence that people are turning toward online sources to find out more about EMA mood monitoring apps. A common pattern was asking for recommendations and sharing preferences (see Additional file 1: Table S1 in Appendix B).

#### What are the barriers to using EMA?

Practical barriers to use by young people were identified by both groups. Both psychologists and young people reported that a lack of motivation to engage and pay attention to feelings, especially when feeling well or without a clear purpose to monitor, was a major barrier to use. Young people also reported that receiving too many EMA prompts and not knowing how to interpret EMA data were foreseeable barriers to use and beneficial outcomes. Psychologist typically raised concerns about technology, including how to deal with technical issues, lack of access to infrastructure, and data privacy issues.

#### Are there any harms? Who might it be most (and least) helpful for?

The key harm reported by young people was that attention to negative emotions, such as feeling low or apathetic, could reinforce those feelings. Two psychologists echoed this concern. In using EMA to measure or increase awareness, young people were concerned that forcing selection between unrepresentative emotion options could make them feel worse, confused, or invalidated. Relatedly, given duty of care responsibilities, psychologists stated that inbuilt connectivity to support services, access to data, and receiving updates about risk would be necessary functions of EMA to keep young people safe.

Both groups identified characteristics of young people that are important to consider when increasing awareness and/or using EMA monitoring. There was some overlap in perspectives. For example, both young people and psychologists identified that individuals who were highly motivated to monitor might benefit most from EMA approaches. Introspective individuals might benefit or be harmed. On the one hand, they might be more able to engage with EMA processes; on the other hand, they might be prone to ruminate about negative experiences—particularly those with anxiety. Young people also identified that younger individuals and those with a clear sense of identity might benefit most from attending to their emotions. Psychologists suggested that individuals who were female, conscientious, and school-aged might benefit most, but those with severe mental health problems might benefit least.

## Discussion

### Can affective awareness and EMA assist in the prevention and/or early intervention of youth depression?

Across data sources, we found converging evidence that affective awareness is important in the prevention and early intervention of depression. Further, EMA can be used as a therapeutic tool to increase awareness. Monitoring mood using EMA does not seem to directly reduce depression but does so indirectly through heightening affective awareness. The indirect effect was found with sub-clinical and clinical samples, suggesting utility for early intervention (and treatment). Stakeholders also indicated that for EMA to have beneficial effects, EMA should focus not just on the *what* but also on the *why* and *how* (i.e., emotional triggers and how to deal with them). This aligns with reports that affective awareness provides a foundation that other skills can leverage and build upon.

None of the studies tested prevention where samples did not have any symptoms and assessed depression over the longer-term. Longitudinal studies are needed to identify new cases and trajectory of symptoms. Young people were also uncertain about preventative effects of affective awareness/EMA due to a lack of perceived need, although psychologists identified links between building awareness from an early age and other cognitive/emotional skills. Our results indicated that a lack of motivation to monitor mood when not symptomatic was likely to be a barrier in the use of EMA for preventive purposes.

All data sources indicated that EMA can be used as a measurement tool to provide rich data about emotional experiences. By detecting intensity and variation in emotions, EMA can identify emotional risk factors for depression as well as predict treatment outcome and course. Young people and psychologists emphasised that use would be determined by characteristics of the individual and desired function. However, overall, the stakeholders were unsure how to access or find appropriate EMA apps. The online forum data, for example, explicitly shows that some people are actively looking for apps and are relying on word-of-mouth for recommendations.

### Individual differences

Individual differences have not been explored comprehensively in the literature. There is some evidence that age moderates the relationship between difficulties with affective awareness and depression [22], and limited evidence for sex differences. In our results, individual differences were linked to the perceived benefits and harms of EMA—with questions around the likelihood and frequency of use, the level of detail in monitoring needed for benefit (i.e., type, intensity, and triggers of emotion), and the type of engagement related to harms. Further, EMA in healthy samples does not seem to increase negative affect over time. An implication is that monitoring for prevention (or early intervention) may not increase distress as predicted by our young and psychologist stakeholders.

Another key finding was that motivation to increase awareness or use EMA might be low when young people are already feeling well. Of studies using a universal approach or healthy samples, one did not report on EMA compliance [33], another reported high EMA compliance (66–100%, 36), and another reported an average compliance rate of 55.4% [39]. The generally low and variable compliance rates are consistent with stakeholder concerns about motivation, but there are likely differences in how motivation plays out in the context of a research trial, clinical practice, and self-directed use.

### Affective awareness is a foundational skill

**Fig. 1:**
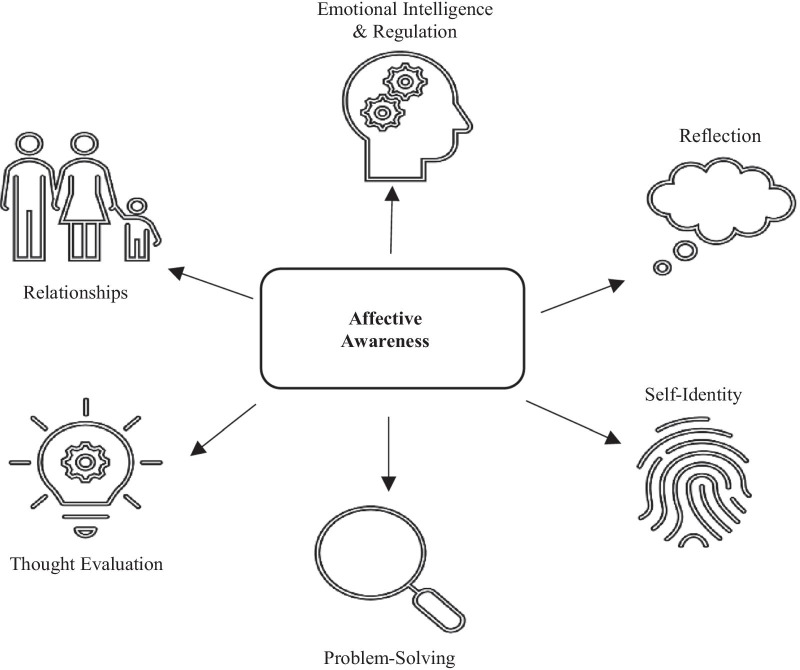
Identified links between active ingredients

The literature and stakeholder perspectives indicate that being aware of feelings facilitates the use of other coping strategies (Fig. 1), and that there is a benefit of embedding affective awareness training into other forms of therapy. Affective awareness is already a common therapeutic process in many psychological interventions for young people and is often introduced as a pre-requisite skill for more complex cognitive and emotion regulation skills. For example, the basic building block for cognitive therapy is the identification of negative thoughts and an understanding of how they impact emotion. An implication is that enhancing awareness may accelerate engagement with existing early intervention programs, enhancing cost-effectiveness in terms of therapeutic outcomes. Further, by using phone-based EMA, more young people can be reached at the same time, with minimal additional costs or resources.

### Proposed mechanisms of action

**Fig. 2:**
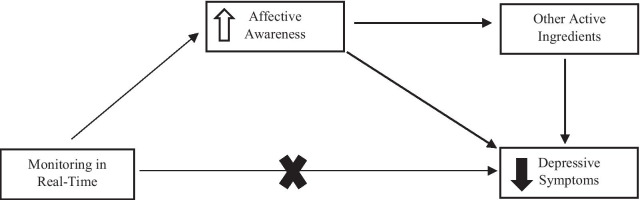
Hypothesised relationship between affective awareness and depression

Our integrated review offers hypotheses about how affective awareness influences depressive symptoms (Fig. 2). First, there might be a direct relationship, whereby increased awareness reduces symptoms (or potentially prevents their occurrence in the first place). Second, there might be an indirect relationship through links with other active ingredients. Increased affective awareness might enable the use of other skills, such as emotional intelligence and regulation, problem solving, thought evaluation, and reflection. It might also help young people to consolidate their identity (by helping them to understand themselves better) and navigate relationships.

### Summary of the knowledge gaps

Phone-based EMA was used almost exclusively in research and less so by young people in their lives. Young people who did use EMA were encouraged by a psychologist, although none of our psychologists reported current integration into routine practice. Whereas research tended to focus on tracking fine-grained variation of emotions, clinical focus was on generating an overall picture of emotional experience to guide treatment. Consistent with prior research, young people were open to mood monitoring, but use depended on function, motivation, and perceived benefits [50]. Key preferences included personalisation, and a simple intuitive design that allows for accurate representation and review of complex moods [51]. A common function underpinning use was linking emotions with triggers and coping skills. This is where research is heading—precision medicine to identify risk factors or vulnerable periods in a young person’s life and to deliver of tailored, just-in-time strategies.

### Future steps

Based on our results, we make the following recommendations:

1. Taking a systematic approach to research efforts by examining: (i) how affective awareness develops over time for different individuals; (ii) how EMA can be used to measure and/or increase affective awareness for different individuals; (iii) the functionality of EMA; and iv) what young people want in an EMA monitoring app, and aligning research efforts with intended use.
2. Evaluating EMA methodologies to identify ideal parameters (e.g., number of daily prompts), with a focus on balancing research rigor with practical relevance.
3. Positioning what young people want and what is done in clinical practice at the forefront of research questions, to increase practical impact for target audiences.
4. Developing publicly available repositories of monitoring apps for young people and psychologists to help them find apps that have been reviewed for quality. An embedded social forum could facilitate personal recommendations.
5. Considering how scalable approaches will address young people’s needs and preferences.
6. Building affective awareness earlier, through childhood learning programs in schools and early education contexts.
7. Exploring how specific components of evidence-based approaches, such as MBCT, influence affective awareness and depressive symptoms in real-time in real-world contexts using EMA.

### Limitations in methodological approach

We used a creative approach to reviewing and interpreting different data sources, positioning lived experiences at the centre of our inquiry to produce meaningful outcomes for young people. However, our sample was limited in some respects. A small number of participants were recruited online using convenience and snowball sampling methodology. An implication of this recruitment strategy is that our sample may not be representative of young people in general or psychologists involved in their care. There are also limits to narrative review methods that warrant mention, such as selection bias, how studies are analysed, and the conclusions drawn [52]. Given the few number of studies that explore EMA and affective awareness in youth depression, and the variation in methodological approaches and quality, a narrative review was better placed to generate meaningful conclusions than a systematic review. Further research using systematic approaches once high-quality evidence has accumulated may corroborate our findings and help to answer different questions about EMA, affective awareness, and youth depression.

## Conclusion

Affective awareness is a foundational skill for young people’s emotional health. EMA can be used as an intervention to increase affective awareness, or as a measurement tool to monitor affective awareness. Initial empirical evidence points to the use of EMA in indicated prevention approaches, and more work is needed in the context of universal and selective prevention. However, psychologists and some young people see benefits in monitoring emotions in real-time before the onset of symptoms, to build a repertoire of emotional skills and, in turn, reduce vulnerability to depression. Affective awareness is likely linked to other cognitive and emotional skills involved in psychological therapy, making it an attractive target to enhance other therapeutic effects.

## Supplementary Information

**Additional file 1.** Contains information about focus group and interview guides (Appendix A), results for publicly available data sources (Appendix B), and additional characteristics about the expert samples (Appendix C).

## Data Availability

The narrative review data are available from the corresponding author upon reasonable request.

## Abbreviations

ACPA: Australian Clinical Psychology Association
BDI-II: Beck Depression Inventory
BSI-AV: Brief Symptom Inventory-Adolescent Version
CBT: Cognitive Behavioural Therapy
DASS-21: Depression Anxiety Stress Scales
DISE: Daily Inventory of Stressful Events
DSM-IV: Diagnostic Statistical Manual, Fourth Edition
EMA: Ecological Momentary Assessment
eMPRac: Black Dog Institute e-Mental Health in Practice
ESA: Emotional Self-Awareness Scale
ESA-R: Emotional Self-Awareness Scale-Revised
FTF: Face-to-Face
GAD-7: Generalised Anxiety Disorder 7
IP: Indicated Prevention
K6: Kessler Scale
K-SADS-PL: Kiddie Schedule for Affective Disorders and Schizophrenia—Present and Lifetime Version
MBCT: Mindfulness-Based Cognitive Therapy
MDD: Major Depressive Disorder
mDES: Modified Differential Emotions Scale
PANAS: Positive and Negative Affect Schedule
PANAS-C: Positive and Negative Affect Schedule for Children
PHQ-9: Patient Health Questionnaire-9
RCT: Randomised Controlled Trial
STEP: Skills to Enhance Positivity Program
T: Treatment
UP: Universal Prevention

## Footnotes

1 Note that while we attempted to recruit young people between the ages of 14–20, only those aged 16 and above expressed interest and signed on to the study.
