# Supplementary material for: Prevention and early intervention of depression in young people: an integrated narrative review of affective awareness and Ecological Momentary Assessment
Source: BMC Psychol. 2021 Aug 16;9:113. doi: 10.1186/s40359-021-00614-6 (PMC8365890; doi:10.1186/s40359-021-00614-6)
Supplement: Supplementary file 1 — Additional file 1. Contains information about focus group and interview guides (Appendix A), results for publicly available data sources (Appendix B), and additional characteristics about the expert samples (Appendix C). [file 40359_2021_614_MOESM1_ESM.docx]

**Appendices**

**Appendix A: Focus Group and Interview Question Guides**

Note. Not all questions were asked for all groups or individuals.

*Focus group question guide*

- Introductions and explanation of focus group procedures

***1) Awareness of emotions and moods***

- What do you all think about emotional awareness?
- In general, how aware do you think you are of how you are feeling?
  - - Have you always been this aware?
    - Are there certain emotions that you are more or less aware of?
    - What do you think has helped/or would help to increase it?
    - So far we’ve spoken about being aware of certain emotions, what about the intensity of those emotions? Emotion variability? Mixed emotions?
- What do you think is the role of emotional awareness in depression?
- For you personally, what are the benefits of being aware of your emotions?
  - Are there any downsides?
- Would anyone like to become more aware of their emotions, as they occur in daily life?
  - What do you think would be a good way to do this?
- What other skills might you gain from having awareness of your moods and emotions?

***2) Understanding and use of mood monitoring***

- What does the term “mood monitoring” mean to you?
  - Where have you heard this term before? What experience do you have with it?
    - [Enquire about psychological therapy if appropriate]**
  - Does anyone currently monitor their mood on a regular basis? What about in the past?
  - For those of you that have:
    - Tell me about how you go/went about it (e.g., automatic or conscious)
    - What do/did you use to do it?
      - Pen-and-paper? Online tools? Smartphone apps?
      - How did the application work/were there any prompts/how long did it go for?
      - What kind of approach worked best for you? Why?
  - What effect does/did mood monitoring have on you?
    - Were there any noticeable effects on your mood over time?
    - Positive/negative?
- For those of you that have not:
  - What has stopped you from mood monitoring?
  - If you were to do it, how would you do it?

***3) Understanding and use of ecological momentary assessment***

- So we’ve spoken a bit about mood monitoring, what do you all know about ecological momentary assessment? [explain if not many people know what it is]
- For those of you that have heard of it before:
  - Tell me a bit about what you know
  - In what way have you personally used it before?
  - What were the benefits of using this technique (e.g., learning more about how emotions fluctuate across the day?)
- In what way do you think something like this could be useful to help young people to understand what they are feeling?
  - What about any potential negative consequences?
  - What level of detail do you think is necessary in EMA – for example, is having a general idea of how you are feeling across the day enough, or are moment-to-moment changes, across minutes or hours, also useful?
  - What would be your optimal balance between awareness and the amount of effort/time you need to spend monitoring?

***4) **Mood monitoring in psychological therapy (if appropriate)***

- If you feel comfortable, I’m wondering if people who have received mood monitoring during psychological treatment could elaborate on their experiences?
  - How was it introduced?
  - How did you and the psychologist interact with the data?
  - When was it used during treatment (at the start, ongoing)?
  - How do you think it influenced your coping strategies?
  - What was your view on how it fits in with the broader purpose of therapy?

***5) Views about depression and gaps in treatment/prevention***

- How do you think that mood monitoring fits in with prevention efforts for depression? What about treatment?
- From your experience, what do you think would help young people to build resilience against low mood or depression?
- What advice would you give to researchers who are working in this space, in terms of what they could think about to improve prevention and early intervention?
- What advice would you give to psychologists or other mental health professionals?

*Interview question guide*

- Introductions and explanation of interview procedures

***1) Awareness of emotions, moods, and depression***

- What does your clinical experience tell you about the role of emotional awareness in depression?
- When young people present for depression or low mood, are there any common patterns you have noticed about how aware they are of what they are feeling?
  - Are there emotions that they are typically more or less aware of?
- How does this level of awareness typically change over the course of treatment?
- How do you typically get a sense of this change over time?
- What other skills might consequently stem from having emotional awareness?

***2) Use of mood monitoring in clinical practice***

- What does the term “mood monitoring” mean to you?
  - How do you typically integrate mood monitoring into treatment?
    - Tell me more about this approach:
      - Pen-and-paper? Online tools? Smartphone apps?
        - [Enquire about online tools and apps if appropriate]
      - How do you select the approach that you use for each young person?
- How does mood monitoring influence the direction of treatment?
- How do you think mood monitoring might be helpful prior to treatment (e.g., before CBT)?
  - How do young people typically respond to mood monitoring?
  - In your experience, what are the characteristics of young people that tend to respond well to or engage with mood monitoring?
    - What do you think are the barriers to positive engagement?

***3) Understanding and use of ecological momentary assessment in psychological therapy***

- So we’ve spoken a bit about mood monitoring, what do you know about ecological momentary assessment? [explain if necessary]
- What is your view of using ecological momentary assessment as part of psychological treatment for young people?
  - Do you use it? What are your reasons for using/not using it?
  - Are you aware of any of your colleagues using it in practice?
- Describe how you could see it integrating with how you normally work with depressed young people
  - What are the difficulties/benefits you anticipate?
- What do you think about young people who are at risk of or have early signs of depression using ecological momentary assessment to get a sense of their emotions without aid from a psychologist?
- Are any unintended consequences of using mood monitoring, whether through EMA or other methods? What are they?
  - How do you think this can be addressed?
- What type of young people do you think would/would not benefit from using mood monitoring/EMA?

***4) Views about depression and gaps in treatment***

- In general, how do you think that mood monitoring fits in with prevention or early intervention efforts for depression? What about the role of emotional awareness?
- From your experience, what do you think would help young people to build resilience against low mood or depression?
- What do you think are the “active ingredients” of psychological therapy for young people presenting for depression?
- What advice would you give to researchers who are working in this space, in terms of what they could think about to improve prevention and early intervention?
- What advice would you give to other psychologists or other mental health professionals?

**Appendix B:** **Results for Publicly Available Data Sources**

Table S1. Summary of publicly available data sources, information extracted, and interpretations.

| **Source** | **Sample/Age** | **Question/ Themes** | **App Preferences** | **Interpretations** |
| --- | --- | --- | --- | --- |
| ***Reddit*** | Undefined | What’s a good mood tracker? **Thread** | Likes: graphing and visualisation, tracking average moods over different timescales (e.g., months, years), simple and easy to use | Daylio is very popular but there are other mood tracking apps that are used. There is not a one-size fits all when it comes to preference. |
|  |  |  | Dislikes: too much focus on tracking and not enough on journaling, entering text on phone |  |
|  |  |  | Apps: eMoods, Daylio, Moodpath, Wysa, Journey, Stories, Mood Log |  |
|  |  | I started an emotion journal today **Thread** | Likes: colours and numbers to represent the complexity of the emotional experience, pictures to represent an emotion experience  Method: pen and paper – colouring in | There are creative ways to represent complex emotions, as well as the intensity of basic emotions. |
| ***Quantifyme.com*** | Undefined | User experiences with mood tracking | Desired features: ability to link moods to activities and locations and collect contextual aspects | Reporting context as well as moods is important. Monitoring increases awareness of emotions/moods, and level of introspection. Monitoring moods and contexts helps users create behavioural and cognitive adjustments that help increase confidence in future situations. |
|  |  |  | Benefits: greater awareness of self, tweaking of everyday life to improve future situations, increased level of introspection, improved confidence |  |
|  |  |  | Apps: Mood Print and Mappiness |  |
| ***Australian Clinical Psychology Association (email listserv) & E-Mental Health in Practice (forum)*** | Adult Mental Health Professionals | What mood monitoring apps do you like/use? | Likes: free, easy, simple, ability to change the names of moods and emotions, notes feature | i-MOOD, Daylio and Mood coach are popular. Professionals want to be able to edit or change the names of emotions/moods. |
|  |  |  | Desired features: preference for tracking other aspects of wellbeing such as contexts, sleep, activity, alcohol intake, workload |  |
|  |  |  | Apps: Mood coach, Daylio, i-Mood journal, myCompass, MindGuage, Moodgym, Moodmission |  |
| **Next Generation Blog** | Audience: young people and parents | Blog post about “7 apps to boost wellbeing” by Justin Coulson, PhD | Apps: Mood Meter, Mindshift, Optimism, My Mood Tracker, Deep Sleep, Depression Check, and Strava  Features: record emotions, moods, and contextual information, provide solutions/strategies for proactive thinking, doctor-patient information sharing  Key outcomes: encourage help-seeking, emotion regulation strategies, relaxation strategies and sleep | There are a variety of apps that encompass at least a small component of mood monitoring. |

**Appendix C: Additional Characteristics of the Expert Samples**

Table S2. Individual characteristics and mood monitoring.

| **Young People** | | **Psychologists** | |
| --- | --- | --- | --- |
| **Measure** | ***n (*%)** | **Measure** | ***n (*%)** |
|  |  |  |  |
| **Born in Australia** |  | **Born in Australia** |  |
| Yes | 18 (75) | Yes | 3 (60) |
| No | 6 (25) | No | 2 (40) |
|  |  |  |  |
| **Gender identity** |  | **Aboriginal or Torres Strait Islander** | 0 |
| Female | 16 (66.7) |  |  |
| Male | 7 (29.2) | **Received training in adolescent mental health** |  |
| Non-binary | 1 (4.2) | Yes | 2 (40) |
|  |  | No | 1 (20) |
| **Aboriginal or Torres Strait Islander** | 0 | Unsure | 2 (40) |
|  |  |  |  |
| **Attend high school** |  | **Recommend mood monitoring as part of treatment for youth depression** |  |
| Yes | 18 (75) | Sometimes | 1 (20) |
| No | 6 (25) | Often | 2 (40) |
|  |  | Always | 2 (40) |
| **Current year group in high school** |  |  |  |
| 10 | 2 (11.8) | **Importance of mood monitoring in psychological treatment** |  |
| 11 | 2 (11.8) | Very important | 1 (20) |
| 12 | 13 (76.5) | Somewhat important | 3 (60) |
|  |  | Unsure | 1 (20) |
| **Currently employed** |  |  |  |
| Yes | 13 (54.2) | **Monitoring results impact therapeutic approach** |  |
| No | 11 (45.8) | Yes | 4 (80) |
|  |  | Unsure | 1 (20) |
| **Mental health diagnosis** |  |  |  |
| Yes | 14 (58.3) | **Use EMA as mood monitoring tool in treatment for youth depression** |  |
| No | 8 (33.3) | Yes | 1 (20) |
| Unsure | 2 (8.3) | No | 4 (80) |
|  |  | No | 4 (80) |
| **Received psychological therapy for depression** |  |  |  |
| Yes | 12 (85.7) |  |  |
| No | 2 (14.3) |  |  |
|  |  |  |  |
| **Used mood monitoring techniques** |  |  |  |
| Yes | 13 (54.2) |  |  |
| No | 11 (45.8) |  |  |
|  |  |  |  |
| **Mood monitoring recommended by a psychologist as part of therapy** |  |  |  |
| Yes | 8 (61.5) |  |  |
| No | 5 (38.5) |  |  |
|  |  |  |  |
| **Open to using mood monitoring in the future** |  |  |  |
| Yes | 21 (100) |  |  |
|  |  |  |  |
